# EID1 overexpression in brown adipose tissue enhances cold-induced thermogenesis in male mice

**DOI:** 10.1016/j.isci.2026.116703

**Published:** 2026-07-11

**Authors:** Itsuki Takahashi, Diana Vargas, Yusuke Watanabe, Tomohiko Sato, Mitsue Miyazaki, Hirofumi Hanaoka, Takahito Nakajima, Ryosuke Kaneko, Izuki Amano, Takeo Nakanishi, Fernando Lizcano, Noriyuki Koibuchi, Noriaki Shimokawa

**Affiliations:** 1 Department of Nutrition, Takasaki University Graduate School of Health and Welfare, Takasaki, Japan; 2 Department of Integrative Physiology, Gunma University Graduate School of Medicine, Maebashi, Japan; 3 Center of Biomedical Investigation (CIBUS), Universidad de La Sabana, Chía, Colombia; 4 Ota College of Medical Technology, Ota, Japan; 5 Department of Bioscience and Laboratory Medicine, Hirosaki University Graduate School of Health Science, Hirosaki, Japan; 6 Near InfraRed Photo-Immunotherapy Research Institute, Kansai Medical University, Hirakata, Japan; 7 Department of Diagnostic and Interventional Radiology, Faculty of Medicine, University of Tsukuba, Tsukuba, Japan; 8 Medical Genetics Research Center, Nara Medical University, Kashihara, Japan; 9 Faculty of Pharmacy, Takasaki University of Health and Welfare, Takasaki, Japan

**Keywords:** EID1, EID1 transgenic mouse, thermogenesis, brown adipose tissue, cold environment

## Abstract

E1A-like inhibitor of differentiation 1 (EID1), a nuclear coregulator, has been implicated in adipocyte differentiation and lipid metabolism. *In vitro*, EID1 overexpression suppresses lipid accumulation in preadipocytes; however, its role in thermogenesis *in vivo* remains unclear. We generated adipose tissue-specific EID1 transgenic (Tg) mice and assessed their thermogenic capacity under cold exposure. During cold exposure, male Tg mice exhibited more than a 7-fold increase in interscapular brown adipose tissue (BAT) glucose uptake, nearly triple the response observed in wild-type (Wt) mice. Core body temperature was better maintained in Tg mice, with attenuated cold-induced decline. Infrared thermography revealed a 5-fold greater increase in BAT temperature in Tg mice than in the Wt controls. These responses were accompanied by robust upregulation of thermogenesis-related genes. These findings highlight EID1 as a dynamic regulator of thermogenic capacity and suggest that modulating its stability and activity may represent a new strategy to improve metabolic health.

## Highlights

• EID1 overexpression enhances cold-induced thermogenesis in adipose tissue
• In EID1 Tg mice, glucose uptake and thermogenic gene expression are increased in BAT
• Core temperature is better maintained in Tg mice during cold stress
• EID1 identified as a dynamic regulator of adipose thermogenic capacity

## Graphical abstract

**Figure:**
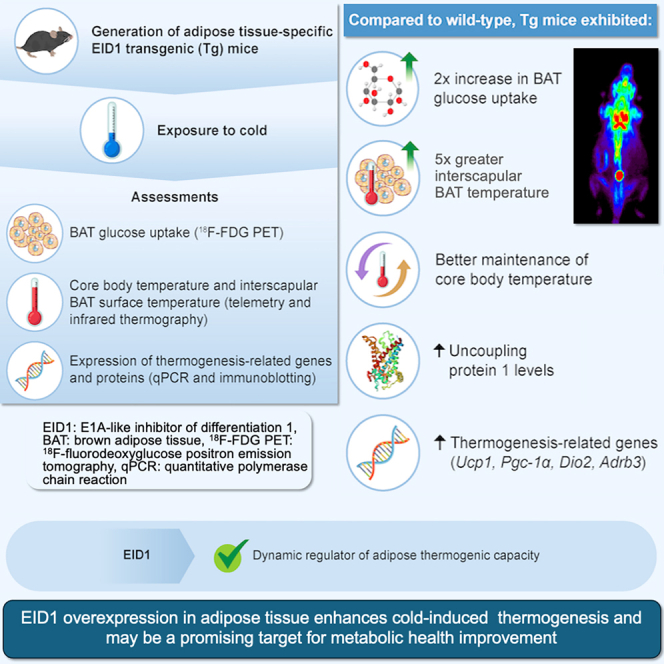

## Teaser

Animal physiology; Biological sciences; Molecular biology

## Introduction

Mammals, including humans, are endothermic and maintain a stable core body temperature through metabolic heat production—a trait that evolved independently in mammals and birds and provides major adaptive advantages.^1^^,^^2^ Thermogenesis occurs via shivering, which involves involuntary muscle contraction, and non-shivering mechanisms, primarily mediated by brown adipose tissue (BAT). BAT is densely vascularized, highly innervated, and rich in mitochondria, enabling efficient heat generation in response to cold. Sympathetic activation during cold exposure (CE) triggers the release of norepinephrine and epinephrine, which bind to the β3-adrenergic receptors (Adrb3) on brown adipocytes. This signaling stimulates lipolysis and mitochondrial heat production through uncoupling protein 1 (UCP1).^3^^,^^4^^,^^5^ UCP1, located in the inner mitochondrial membrane, dissipates the proton gradient generated by the electron transport chain as heat rather than coupling it to ATP synthesis, thereby increasing energy expenditure and contributing to cold tolerance and protection against obesity.^6^^,^^7^^,^^8^

BAT was once believed to be restricted to small mammal species and human newborns; however, ^18^F-fluorodeoxyglucose positron emission tomography (^18^FDG-PET) revealed metabolically active UCP1-expressing BAT depots in adults in the cervical, supraclavicular, and paraspinal regions.^9^ This “rediscovery” challenged the long-held assumption of BAT’s absence in adults and reinvigorated interest in its potential metabolic role. It also prompted the hypothesis that activating BAT could increase whole-body energy expenditure, enhance glucose and lipid utilization, and thereby provide therapeutic benefits in metabolic disorders such as obesity, fatty liver disease, and type 2 diabetes. Consequently, substantial research has focused on identifying molecular targets and pharmacological agents capable of activating BAT or recruiting thermogenic adipocytes.^10^ Within this context, we have focused on the E1A-like inhibitor of differentiation 1 (EID1), a nuclear protein originally identified for its interactions with the retinoblastoma protein (pRb), p300, and CREB-binding protein (CBP), through which it suppresses cell differentiation.^11^^,^^12^^,^^13^^,^^14^ Beyond its role in cell cycle regulation, EID1 has been implicated in adipocyte metabolism. In our previous work using human subcutaneous adipose-derived mesenchymal stem cells and murine preadipocyte cell lines, EID1 overexpression reduced triglyceride accumulation and upregulated thermogenic genes, including *Ucp1* and *Pparγ co-activator 1 α* (PGC-1α).^15^^,^^16^ Pgc-1α is a well-established coactivator that promotes mitochondrial biogenesis and enhances thermogenic capacity in adipocytes. More recently, we demonstrated that EID1 localizes to the nucleus and represses glycerol-3-phosphate dehydrogenase (GPDH), thereby reducing lipid synthesis.^17^ In this study, we aimed to elucidate the functions of EID1 in adipose tissue *in vivo*. Previous *in vitro* findings led us to hypothesize that EID1 may regulate thermogenesis through BAT-associated pathways *in vivo*.

To test this hypothesis, we generated adipose-specific EID1 transgenic (Tg) mice and investigated their thermogenic and metabolic responses to CE, with a particular focus on BAT activity, core body temperature regulation, glucose uptake, and the expression of thermogenesis-related genes to clarify its physiological significance.

## Results

### EID1 transgenic mice overexpress EID1 in adipose tissues

**Figure 1:**
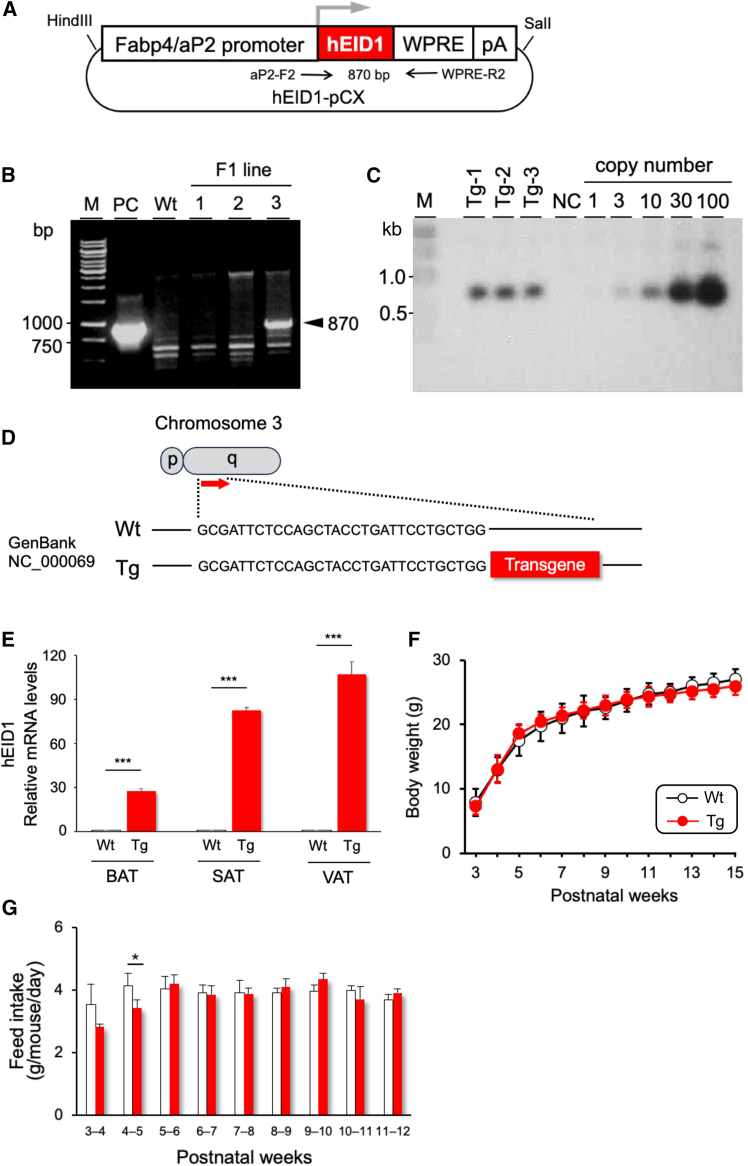
Transgene structure and EID1 expression in transgenic mice (A) Structure of the transgene used in this study. Human EID1 cDNA, ligated to the *Fabp4/aP2* promoter (upstream) and WPRE (downstream), was subcloned into the pCX plasmid. The arrow indicates the translation start site (ATG) of EID1. Lateral arrows denote the primer positions used for genotyping. (B) PCR confirmation of transgenic events. Line 3 of the F1 offspring produced the expected 870 bp product, while lines 1 and 2 showed no amplification, consistent with wild-type (Wt) mice. M: DNA marker; PC: positive control. (C) Southern blot analysis confirms transgene integration. Genomic DNA from three Tg mice (Tg-1, -2, -3) was digested with *Dra*I and hybridized with a ^32^P-labeled probe corresponding to the EID1 region of the transgene. Standards containing 1, 3, 10, and 30 copies were included. Wt genomic DNA served as a negative control (NC). (D) Insertion site of the transgene on mouse chromosome 3. The EID1 transgene localized to the long arm (q). p: short arm; NC_000069: GenBank RefSeq accession number. (E) mRNA expression of EID1 in adipose depots of Wt and Tg mice. Expression in BAT, SAT, and VAT of Wt (□) and Tg () mice was quantified by real-time PCR and normalized to *Gapdh*. Data are expressed as the mean ± SD (*n* = 5). ∗∗∗*p* < 0.001 vs. Wt. (F) Effect of EID1 transgene expression on body weight. Body weight was measured weekly from 3 to 15 weeks of age. Data are expressed as the mean ± SD (*n* = 5). Wt (○) and Tg (). (G) Effect of EID1 transgene expression on feed intake. The quantity of feed intake was measured daily from 3 to 12 weeks of age. The average daily feed intake per mouse is shown in bar graphs for each week. Data are expressed as the mean ± SD. ∗*p* < 0.05 vs. Wt at the same period. Wt (*n* = 5, □) and Tg (*n* = 4, ). Abbreviations: EID1, E1A-like inhibitor of differentiation 1; BAT, brown adipose tissue; SAT, subcutaneous adipose tissue; VAT, visceral adipose tissue; SAT, subcutaneous adipose tissue; Wt, wild-type; Tg, transgenic; WPRE, woodchuck hepatitis virus post-transcriptional regulatory element; Gapdh, glyceraldehyde-3-phosphate dehydrogenase; PCR, polymerase chain reaction; SD, standard deviation.

Southern blotting and rapid amplification of integration sites without interference by genomic repeats (RAISING) analyses confirmed successful integration of the EID1 transgene, present in tandem arrays of >10 and <30 copies on the long arm (q) of chromosome 3 (Figures 1C and 1D). As expected, *EID1* mRNA levels were markedly elevated in all the adipose depots examined—BAT, subcutaneous adipose tissue (SAT), and visceral adipose tissue (VAT)—in Tg mice compared with the wild-type (Wt) controls (Figure 1E). Notably, this robust overexpression was confined to adipose tissues, as no differences were observed in postnatal body weight (Figure 1F). Furthermore, immediately after weaning, Wt mice tended to consume more feed than Tg mice, but no significant difference was observed thereafter (Figure 1G). These findings confirm that the model achieved targeted EID1 overexpression without affecting overall growth.

### EID1 enhances glucose uptake in BAT during cold exposure

**Figure 2:**
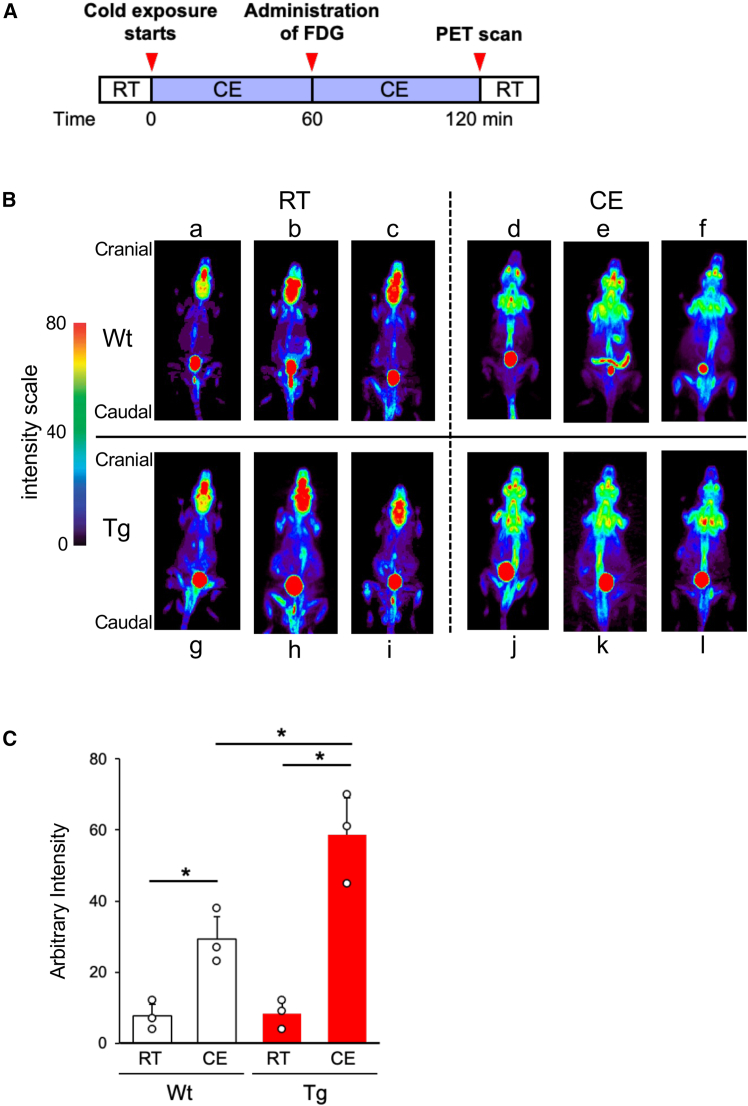
Glucose uptake in adipose tissue of EID1 transgenic mice during cold exposure (A) Experimental schedule. Mice were exposed to cold (4 °C) for 1 h, injected with ^18^F-FDG (5 MBq/mouse, tail vein), and maintained for an additional 1 h at the same temperature before PET scanning. RT: room temperature (25 °C); CE: cold exposure (4 °C). (B) Representative ^18^F-FDG PET images. Upper panel: RT (A–C) and CE (D–F) in Wt mice. Lower panel: RT (G–I) and CE (J–L) in Tg mice. The intensity scale is shown on the left; values are arbitrary. (C) Quantification of ^18^F-FDG uptake in interscapular BAT. Uptake values are expressed as arbitrary units based on the intensity scale. Data are expressed as the mean ± SD (*n* = 3 per group). Symbols: Wt (□), Tg (), individual values (○). ∗*p* < 0.05. Abbreviations: EID1, E1A-like inhibitor of differentiation 1; ^18^F-FDG, ^18^F-fluorodeoxyglucose; PET, positron emission tomography; RT, room temperature; CE, cold exposure; BAT, brown adipose tissue; Wt, wild-type; Tg, transgenic; SD, standard deviation.

At room temperature (RT) (25 °C), ^18^F-FDG PET imaging revealed a similar tracer distribution in both genotypes, with high brain uptake and minimal BAT signals (Figure 2B, RT). By contrast, CE (4 °C, 2 h) markedly reduced brain uptake and, notably, revealed a pronounced interscapular signal corresponding to BAT activation (Figure 2B, CE). Quantitative analysis showed that CE increased ^18^F-FDG uptake in BAT by 3.82-fold in Wt mice and by 7.04-fold in Tg mice. Importantly, this relative increase was significantly greater in Tg mice (*p* < 0.036; Figure 2C), indicating an enhanced metabolic response to cold stimulation.

### EID1 enhances cold-induced thermogenesis

**Figure 3:**
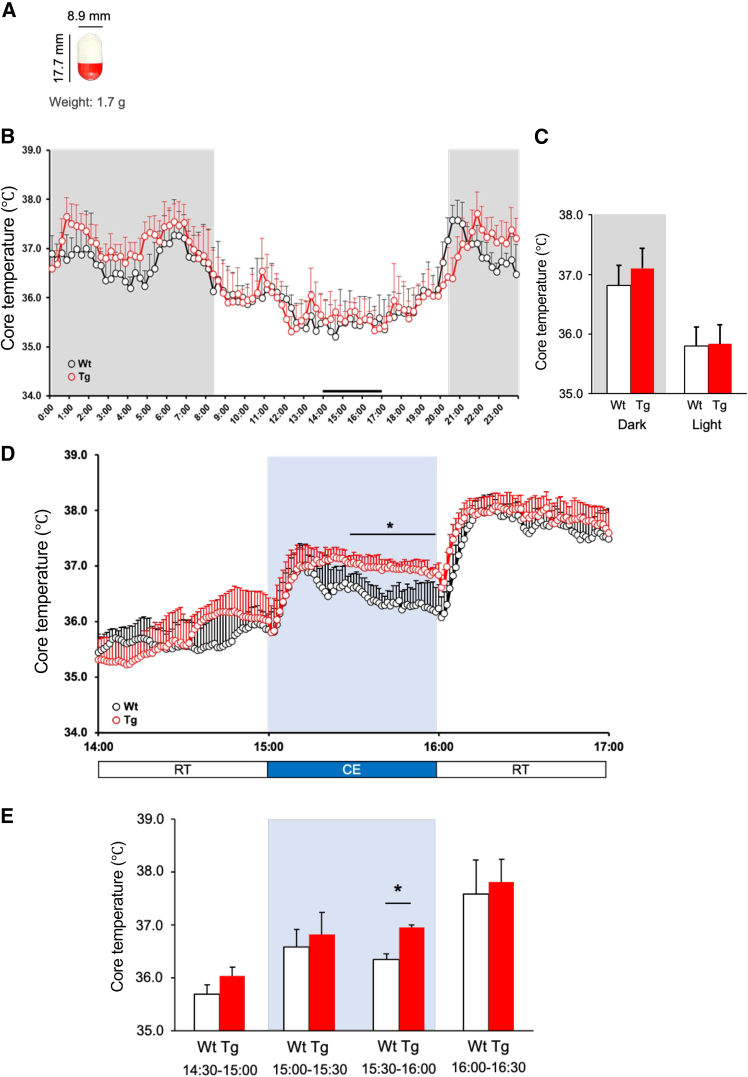
Core body temperature of EID1 transgenic mice during cold exposure (A) Ani pill telemetry capsule used for continuous measurement of core body temperature. (B) Circadian changes in core body temperature. Measurements were obtained from Wt (○) and Tg () mice using implanted telemetry capsules. Data are expressed as the mean ± SD (*n* = 5), recorded every 15 min over a 24-h period. Gray shaded region () indicates the dark phase. The bar highlights the period of least variation (14:00 to 17:00). (C) Average core body temperature during light and dark phases. Wt (□) and Tg () mice were monitored every 15 min across 12-h dark and light periods. Data are expressed as the mean ± SD (*n* = 48). The gray shaded region () indicates the dark phase. (D) Changes in core body temperature during cold exposure. Wt (○) and Tg () mice were exposed to 4 °C between 15:00 and 16:00. Data are expressed as the mean ± SD (*n* = 5) recorded every min from 14:00 to 17:00. The blue shaded region () indicates cold exposure. ∗*p* < 0.05 vs. Wt at the same time point. (E) Average core body temperature of Wt (□) and Tg () mice before, during, and after cold exposure. Data were obtained every min and averaged over 30-min intervals: pre-exposure (14:30–15:00), early exposure (15:00–15:30), late exposure (15:30–16:00), and post-exposure (16:00–16:30). Data are expressed as the mean ± SD (*n* = 30). The blue shaded region () indicates cold exposure. ∗*p* < 0.05 vs. Wt at the same interval. Abbreviations: EID1, E1A-like inhibitor of differentiation 1; Wt, wild-type; Tg, transgenic; SD, standard deviation.

**Figure 4:**
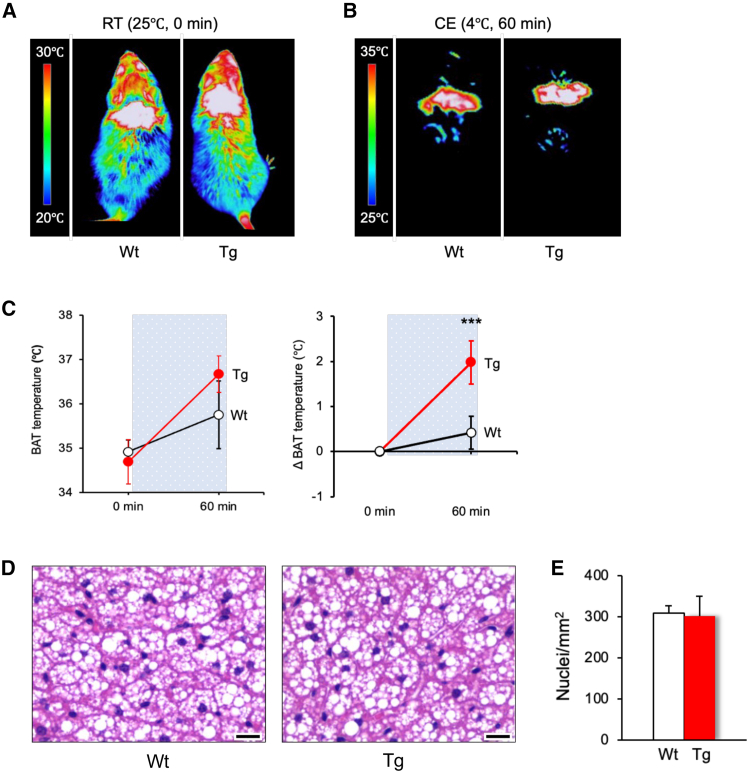
BAT surface temperature of EID1 transgenic mice during cold exposure (A) Representative infrared thermographic image of BAT surface temperature at room temperature (RT, 25 °C). (B) Representative infrared thermographic image after 60 min of cold exposure (CE, 4 °C). The body temperature scale is shown on the left (FLIR Research Studio v2024.5.0). (C) Quantification of BAT surface temperature (left panel) and temperature increment (ΔBAT, right panel) before (RT, 0 min) and after CE (4 °C, 60 min). The blue shaded region () indicates the duration of the cold exposure. Wt (○); Tg (). Data are expressed as the mean ± SD (*n* = 5). ∗∗∗*p* < 0.001 vs. Wt mice at the same time point. (D) Observation of brown adipocytes in BAT by histochemistry. The tissue sections of BAT in Wt (left panel) and Tg (right panel) mice were stained with hematoxylin and eosin (H&E stain). Bar, 50 μm. (E) Average nucleus number in BAT. The number of nuclei in BAT of Wt (□) and Tg () mice was counted in five arbitrary areas of 1 mm^2^ per mouse. Data are expressed as the mean ± SEM (*n* = 4). Abbreviations: EID1, E1A-like inhibitor of differentiation 1; BAT, brown adipose tissue; Wt, wild-type; Tg, transgenic; RT, room temperature; CE, cold exposure; SD, standard deviation.

Continuous telemetry monitoring (via implanted telemetry capsules; Figure 3A) revealed comparable circadian profiles of core body temperature in Wt and Tg mice at 25 °C, with both groups maintaining ∼1.0°C–1.3 °C higher values during the dark phase (Figures 3B and 3C). This stability allowed CE to be initiated at 15:00 h, when baseline temperatures were steady. In response to acute cold, both groups exhibited a rapid rise from ∼35.8 °C to ∼37.0 °C within 10 min. Thereafter, Wt mice displayed a gradual decline to ∼36.0°C by 60 min, whereas Tg mice maintained significantly higher temperatures, reaching ∼36.8 °C at 60 min (Figures 3D and 3E). Genotype-dependent differences became evident from 30 min onward, demonstrating superior thermal maintenance in Tg mice. Infrared thermography corroborated these findings. At 25 °C, interscapular BAT surface temperatures did not differ between groups (Figures 4A and 4C). However, CE induced a substantial increase (ΔBAT) of 2.0 ± 0.20 °C in Tg mice, compared with only 0.4 ± 0.15 °C in Wt controls (*p* < 0.01; Figures 4B and 4C).

To determine whether this substantial increase in temperature in BAT was accompanied by structural changes in the brown adipocytes themselves, we performed hematoxylin and eosin (H&E) staining of BAT. There was no difference in the size or shape of brown adipocytes between Wt and Tg (Figure 4D). To determine the number of cells per unit area (mm^2^), we counted the nuclei; however, no difference was observed between Wt and Tg (Wt: 308 ± 18.8 nuclei/mm^2^, Tg: 302 ± 47.9 nuclei/mm^2^, *p* = 0.89) (Figure 4E).

Together, these physiological data indicate that EID1 overexpression enhances the capacity of BAT to sustain core body temperature during thermal stress, consistent with the observed increases in glucose utilization.

### EID1 upregulates the expression of thermogenetic genes in BAT during cold exposure

**Figure 5:**
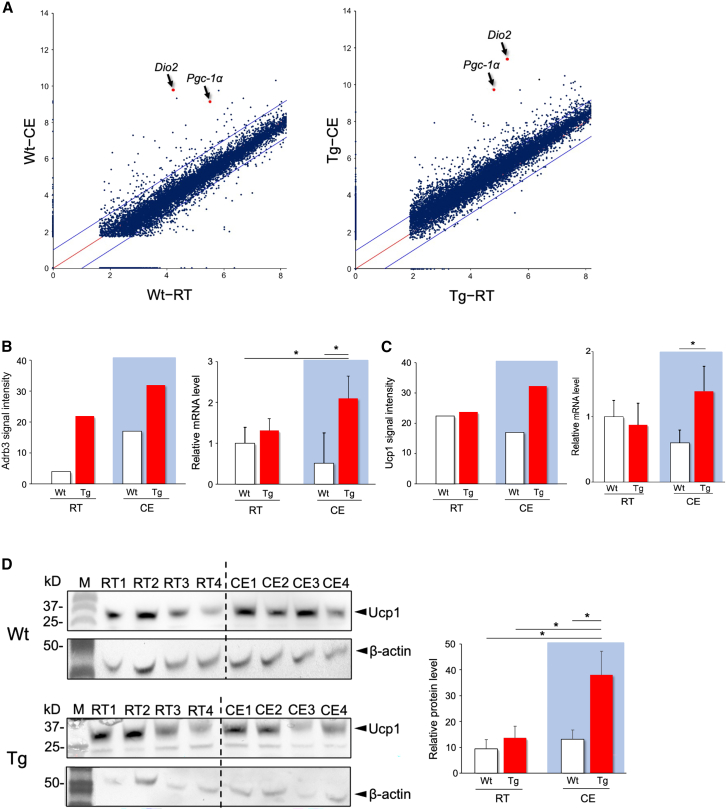
Expression of thermogenesis-related genes and proteins in EID1 transgenic mice (A) mRNA expression levels of thermogenesis-related genes in BAT of Wt (left panel) and Tg (right panel) mice at RT and after cold exposure (CE) were analyzed by DNA microarray. The red and blue lines represent y = x and ±2-fold fluctuation range, respectively. Red dots () indicate *Pgc-1α* and *Dio2*, which were strongly upregulated by cold exposure. (B) Expression of *Adrb3* in BAT. Left panel: DNA microarray analysis. Right panel: qRT-PCR. Data for qRT-PCR are expressed as the mean ± SD (*n* = 5). The blue shaded region () indicates cold exposure (4 °C, 60 min). Symbols: Wt (□), Tg (). ∗*p* < 0.05. (C) Expression of *Ucp1* in BAT. Left panel: DNA microarray analysis. Center panel: qRT-PCR. Right panel: western blotting. Data for qRT-PCR and western blotting are expressed as mean ± SD (*n* = 5). The blue shaded region () indicates cold exposure (4 °C, 60 min). Symbols: Wt (□), Tg (). ∗*p* < 0.05. (D) UCP1 protein expression levels in BAT. Left panel: representative immunoblots of UCP1 in Wt (upper) and Tg (lower) mice under RT and CE (*n* = 4 each). Molecular mass markers (M, kDa) are shown on the left. Right panel: densitometric quantification of UCP1 protein normalized to β-actin. The blue shaded region () indicates cold exposure (4 °C for 60 min). Data are expressed as the mean ± SD (*n* = 4). ∗*p* < 0.05 vs. Wt mice (□) and vs. RT in Tg mice (). Abbreviations*:* EID1, E1A-like inhibitor of differentiation 1; BAT, brown adipose tissue; Wt, wild-type; Tg, transgenic; RT, room temperature; CE, cold exposure; mRNA, messenger RNA; qRT-PCR, quantitative reverse transcription-polymerase chain reaction; SD, standard deviation.

Gene expression profiling of BAT after 1 h of CE revealed the robust activation of thermogenic pathways. *Pgc-1α* mRNA levels increased 12.1-fold in Wt mice and 29.0-fold in Tg mice (Figure 5A), while iodothyronine deiodinase 2 (*Dio2*) expression rose 18.5-fold and 36.5-fold, respectively. It is well established that CE upregulates the expression of these two genes in BAT.^18^^,^^19^ However, the extent of this increase is higher in Tg than in Wt, with 2.4-fold and 2-fold higher increases observed for *Pgc-1α* and *Dio2*, respectively. Microarray analysis also identified upregulation of *Adrb3* and *Ucp1*, which was confirmed by qRT-PCR (Figures 5B and 5C). Consistent with the transcriptional data, UCP1 protein abundance—a central effector of BAT thermogenesis—was significantly elevated in Tg mice (Figure 5D). Collectively, these molecular findings demonstrate that EID1 overexpression not only amplifies cold-induced BAT activation but also strengthens critical regulatory nodes of the thermogenic program.

## Discussion

Our findings demonstrate that adipose-specific overexpression of EID1 enhances thermogenic capacity in male mice *in vivo*, as evidenced by increased glucose uptake in BAT, improved maintenance of core body temperature during cold stress, and robust induction of thermogenesis-related genes such as *Ucp1* and *Pgc-1α*. Notably, ^18^F-FDG PET revealed that glucose uptake in BAT increased more than 7-fold in Tg mice during CE—nearly triple the response observed in Wt mice. This difference indicates that EID1 augments substrate utilization in BAT beyond the physiological response to cold. Consistently, Tg mice maintained higher core body temperatures under cold stress, with a markedly attenuated decline compared with Wt controls. Infrared thermography further supported this phenotype, demonstrating a 5-fold greater increase in interscapular BAT surface temperature in Tg mice.

**Figure 6:**
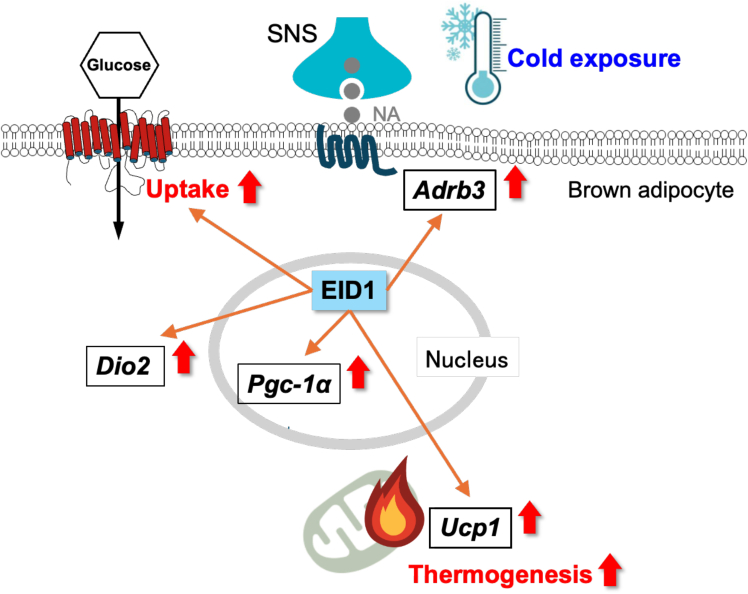
Proposed model of molecular signaling underlying thermogenesis in BAT of EID1 transgenic mice during cold exposure Cold exposure enhances glucose uptake in brown adipocytes of EID1 Tg mice, which is utilized for thermogenesis. Overexpression of EID1 promotes the expression of *Adrb3*, *Dio2*, *Pgc-1α* and *Ucp1*, leading to increased thermogenic activity and maintenance of body temperature. Abbreviations Adrb3, β3-adrenergic receptor; Dio2, iodothyronine deiodinase 2; EID1, E1A-like inhibitor of differentiation 1; NA, norepinephrine; Pgc-1α, peroxisome proliferator-activated receptor-γ coactivator-1α; SNS, sympathetic nervous system; Ucp1, uncoupling protein 1.

At the molecular level, these physiological and imaging data were accompanied by upregulation of thermogenesis-related genes, including *Pgc-1α*, *Dio2*, *Adrb3*, and *Ucp1*, as well as increased glucose uptake and UCP1 protein abundance. Together, these convergent results strongly support the conclusion that EID1 amplifies BAT activation at metabolic, physiological, and molecular levels (Figure 6). Nevertheless, certain caveats must be acknowledged. ^18^F-FDG PET measures glucose uptake but does not directly capture fatty acid oxidation, the primary substrate for BAT thermogenesis. Moreover, whether the enhanced BAT activity observed in Tg mice translates into improvements in systemic glucose tolerance or whole-body energy balance remains to be determined.

By providing direct *in vivo* evidence that EID1 modulates thermogenesis, our results extend previous *in vitro* observations in mouse cell lines and human adipocytes, in which EID1 was shown to reduce triglyceride accumulation and promote thermogenic gene expression.^15^^,^^16^ Mechanistically, EID1 is a nuclear coregulator with a remarkably short half-life (∼30 min under basal conditions) due to rapid ubiquitin-proteasome degradation.^20^^,^^21^^,^^22^ This instability suggests that EID1 functions as a highly dynamic regulator, allowing rapid adaptation to environmental cues. Recognition by the F box protein FBXO21, which recruits EID1 to the SCF E3 ligase complex, and phosphorylation of specific serine residues appear to mediate its degradation, implying that upstream signaling pathways may dynamically modulate EID1 stability.^23^ In BAT, sympathetic activation during CE could prolong EID1 stability, thereby amplifying its transcriptional effects on thermogenic programs.

An additional regulatory layer may involve protein S-palmitoylation, a reversible lipid modification that controls protein localization, stability, and activity.^24^ Palmitoylation has been reported to modify EID1, influencing its degradation and coordinating CBP/p300 histone acetyltransferase activity during cell differentiation.^25^ We hypothesize that in adipose tissue, cold or β3-adrenergic stimulation may modulate EID1 palmitoylation, transiently extending its half-life and strengthening its transcriptional effects on the *Ucp1/PGC-1α* axis. Such a mechanism could provide a molecular explanation for the enhanced thermogenic phenotype observed in Tg mice.

However, this study was unable to provide experimental evidence to demonstrate an association between EID1 and Ucp1/Pgc-1α or the Adrb3 pathway. In future studies, we intend to investigate the EID1-pRb-thermogenesis axis. pRb binds to EID1 as a factor that drives Ucp1, Pgc-1α, and Adrb3 toward thermogenesis.^11^^,^^15^ pRb is a key regulator of the differentiation of white adipose tissue—the primary energy reservoir—and BAT, a powerful energy-consuming source via thermogenesis. To date, it has been reported that when embryonic stem cells of Wt and Rb-deficient (Rb^−/-^) mice are induced to differentiate into adipocytes, Ucp1 is exclusively expressed in Rb^−/-^ adipocytes.^26^^,^^27^ In addition, Rb^−/-^ adipocytes have higher expression levels of Pgc-1α.

Another intriguing implication is that EID1 may influence not only classical BAT but also the recruitment of beige adipocytes from white fat depots. In humans, metabolically active depots often comprise mixed brown and beige adipocytes, and beige adipocytes can be recruited from subcutaneous white adipose tissue in response to stimuli such as CE, β3-adrenergic activation, and endocrine factors including fibroblast growth factor 21 and natriuretic peptides. Given EID1’s ability to enhance *Ucp1/PGC-1α* expression while repressing lipogenic genes, its modulation could lower the threshold for adipocyte beiging in human adipose tissue, thereby broadening its translational relevance.^28^^,^^29^

From a clinical perspective, enhancing thermogenic capacity in adipose tissue could, in principle, increase whole-body energy expenditure and improve glucose homeostasis, with potential applications in obesity, type 2 diabetes, and related metabolic disorders. However, given EID1’s known interactions with pRb, p300, and CBP, careful consideration of tissue specificity and temporal control will be critical to minimize the potential off-target or oncogenic effects. Selective strategies may include adipose-targeted gene delivery, small molecules that disrupt E3 ligase recognition, or interventions that transiently stabilize EID1 in response to metabolic cues. The possibility of regulating EID1 stability via S-palmitoylation introduces an additional therapeutic angle. If transient stabilization of EID1 enhances beige adipocyte recruitment and BAT thermogenesis, targeted manipulation of this pathway could represent a new means of improving metabolic health. Nevertheless, rigorous control of tissue specificity and timing will be essential for safe clinical translation.

In conclusion, our study identifies EID1 as a regulator of adipose thermogenesis capable of enhancing glucose uptake and supporting core body temperature through induction of thermogenesis-related genes. Its short half-life and proteasome-mediated turnover suggest that EID1 functions as a highly dynamic regulator that responds to physiological cues. The observed effects also raise the possibility that EID1 may influence not only classical BAT but also the recruitment of beige adipocytes, a process with relevance to human metabolic physiology.

These findings underscore the need to further investigate the molecular mechanisms controlling EID1 stability and activity, including post-translational regulation by ubiquitination and palmitoylation, and to clarify its role in the interplay between energy storage and expenditure across adipose depots. These approaches will be critical for determining whether modulating EID1 holds translational potential to improve metabolic health, while simultaneously advancing our understanding of the molecular regulation of thermogenesis in mammals.

### Limitations of the study

Some limitations should be acknowledged. This study relied on a single Tg overexpression model, and reciprocal loss-of-function approaches—such as adipose-specific EID1 knockout models—are needed to establish causality. The effects of EID1 on thermogenesis should also be evaluated under thermoneutral conditions, which more closely approximate human physiology, and in experimental systems incorporating lineage tracing to distinguish effects on classical BAT versus beige adipocytes. In addition, it is important to determine whether the modulation of EID1 influences cardiovascular function, particularly under conditions of sustained thermogenic activation.

## Resource availability

### Lead contact

Further information and requests for resources should be directed to and will be fulfilled by the lead contact Noriaki Shimokawa (shimokawa-n@takasaki-u.ac.jp).

### Materials availability

Mouse lines generated in this study are available from the lead contact upon reasonable request. Regarding reagents, this study did not generate new unique reagents.

### Data and code availability

All data reported in this paper will be shared by the lead contact upon reasonable request. Regarding code availability, this paper does not report original code to an official platform. However, some of them are available in this paper’s supplemental information, key resources table. Any additional information required to reanalyze the data reported in this paper is available from the lead contact upon reasonable request.

## Acknowledgments

This study was supported by the Grants-in-Aid for Scientific Research (10.13039/501100001691KAKENHI) of 10.13039/501100001691Japan Society for the Promotion of Science [grant nos. 26670457 to N.K. and 22K11722 to M.M.] and a grant from the 10.13039/100032510Takasaki University of Health and Welfare to T.N. and N.S.

## Author contributions

I.T.: conceptualization, investigation, visualization, data curation, formal analysis, and writing – original draft. D.V.: conceptualization, investigation, visualization, and writing – review and editing. Y.W.: investigation. T.S.: investigation and resources. M.M.: investigation and funding acquisition. H.H.: investigation and visualization. T.N.: investigation and visualization. R.K.: conceptualization, investigation, and resources. I.A.: investigation and resources. T.N.: conceptualization, writing – review and editing, and funding acquisition. F.L.: conceptualization, writing – review and editing, funding acquisition, and supervision. N.K.: conceptualization, writing – review and editing, funding acquisition, and supervision. N.S.: conceptualization, investigation, resources, visualization, data curation, writing – review and editing, funding acquisition, and supervision.

## Declaration of interests

The authors declare no competing interests.

## STAR★Methods

### Key resources table

**Table:** 

REAGENT or RESOURCE	SOURCE	IDENTIFIER
**Antibodies**		

Rabbit polyclonal anti-UCP1	Abcam	Cat# ab10983, RRID:AB_2241462
Rabbit polyclonal anti-β-actin	Cell Signaling Technology	Cat#4967, RRID: AB_330288
ECL Rabbit IgG, HRP-linked whole Ab	Cytiva Amersham	Cat# NA934

**Biological samples**		

Mouse brown adipose tissue	This paper	N/A

**Chemicals, peptides, and recombinant proteins**		

^18^F-fluorodeoxyglucose	Gunma University Hospital, Gunma, Japan	N/A
PMSG: Serotropin®	ASKA Animal health	sero_202304.pdf
hCG: Gonatropin®	ASKA Animal health	gona_202304.pdf
dCTP, [α-^32^P]	PerkinElmer	Cat# BLU013H
CelLytic MT buffer	Sigma-Aldrich	Cat# C3228-50 ML

**Critical commercial assays**		

Total RNA extraction kit: RNeasy Plus Universal kit	QIAGEN	Cat#/ID. 73404
Reverse transcription: High-Capacity RNA-to-cDNA kit	Applied Biosystems	Cat# 4387406
Western Blotting Detection Reagent: ECL® Prime	Cytiva Amersham	Cat# 45-002-401

**Deposited data**		

GRCm39 reference genome	NCBI	https://www.ncbi.nlm.nih.gov/datasets/genome/GCF_000001635.27/

**Experimental models: Organisms/strains**		

Mouse: EID1 transgenic mouse	This paper	N/A
Mouse: C57BL/6JmsSlc	Japan SLC	http://www.jslc.co.jp/english/animals/mouse.php#mouse-cat-02

**Oligonucleotides**		

Primer: aP2/WPRE Forward: CTCATAGCACCCTCCTGTG	This paper	N/A
Primer: aP2/WPRE Reverse: GAGGGGGAAAGCGAAAGTC	This paper	N/A
Mouse Eid1 (Mn01337661_s1)	Applied Biosystems	Cat# 4351372
Human Eid1 (Hs00534885_s1)	Applied Biosystems	Cat# 4331182
Ucp1 (Mm01244861_m1)	Applied Biosystems	Cat# 4331182
Adrb3 (Mm02601819_g1)	Applied Biosystems	Cat# 4331182
Pparγ (Mm00440940_m1)	Applied Biosystems	Cat# 4331182
Pgc-1α (Mm01208835)	Applied Biosystems	Cat# 4331182
Gapdh (Mm99999915_g1)	Applied Biosystems	Cat# 4448485

**Recombinant DNA**		

Fabp4/aP2 promoter	Rival et al.^27^	N/A
WPRE	Donello et al.^28^	N/A
hEID1-pCX	This paper	N/A

**Software and algorithms**		

FLIR thermal imaging camera	FLIR tools	RRID:SCR_016330
ImageJ Software	National Institute of Health	https://imagej.net/ij/
OSEM3D/MAP algorithm	Siemens	micro-PET Manager 2.4.1.1
Statistical analyses: EZR software v1.60	Kanda Y.^30^	https://doi.org/10.1038/bmt2012.244
Image analysis software: CS Analyzer 4	ATTO	https://www.attoeng.com/cs-analyzer4
FLIR Research Studio v2024.5.0 software	Teledyne FLIR	https://www.flir.com/discover/rd-science/flir-research-studio-essential-updates/

**Other**		

Inveon animal PET system	Siemens	INVEON_04.pdf
DSI Anipill® telemetry system	BodyCAP	https://www.animals-monitoring.com
Infrared Thermal Camera: FLIR T530	Teledyne FLIR	https://www.flir.com/products/t530/
DNA microarray: 3D-Gene® Mouse Oligo chip 24k	TORAY	https://www.3d-gene.com/en/

### Experimental model and study participant details

#### Mice

EID1 Tg mice were generated using standard pronuclear microinjection techniques.^31^ Fertilized eggs were obtained from superovulated C57BL/6J females treated with pregnant mare serum gonadotropin (PMSG) and human chorionic gonadotropin (hCG), followed by injection of purified transgene DNA. A total of 240 embryos were transferred into the oviducts of eight pseudopregnant foster mothers (∼30 oocytes each). Three of the 25 offspring (12%) carried the EID1 transgene (F0 founders). Founders were bred with Wt mice to establish a stable line (EID1 Tg strain). Genotyping was performed on tail DNA using PCR with primers aP2-F2 (5′-CTCATAGCACCCTCCTGTG-3′) and WPRE-R2 (5′-GAGGGGGAAAGCGAAAGTC-3′) (Figure 1B).

EID1 Tg and Wt mice were bred at the Animal Facility of Takasaki University. C57BL/6J mice (Japan SLC Inc., Hamamatsu, Japan) were used as Wt controls. All animal procedures were approved by the Animal Care and Experimentation Committee of Takasaki University (Approval No. 2105). Mice were maintained under standard laboratory conditions (12:12 h light–dark cycle, lights on at 08:00; 25 ± 1 °C; 55% relative humidity) with free access to water and standard chow. Each morning (08:00), the body weight of each mouse was measured (Figure 1F). Simultaneously, a fixed quantity of feed was provided, and the remaining feed was weighed the following morning to determine intake.^32^ The average daily feed intake per mouse for Wt (*n* = 5) and Tg (*n* = 4) groups is shown in bar graphs for each week (Figure 1G). We used male mice in this study. The reason is that it allows for the exclusion of variations in thermogenesis caused by the estrous cycle in female mice.^33^ Furthermore, in this study, we primarily used 10-month-old mice. The age range of 9–12 months in mice corresponds to middle age in humans.^34^ It has been reported that during this period age-related changes in metabolic function begin to appear in adipose tissue, including adipocyte hypertrophy, increased inflammation, and a decline in the thermogenic capacity of BAT.^30^^,^^35^ Since these changes have a significant impact on lipid metabolism and body temperature regulation, 10-month-old mice were used in this study to evaluate the function of BAT.

### Method details

#### Construction of targeting vector

A schematic representation of the targeting vector is shown in Figure 1A. To achieve adipose tissue-specific expression of EID1, a 5.55 kb *Fabp4/aP2* promoter^36^ was positioned upstream of the gene. The woodchuck hepatitis virus post-transcriptional regulatory element (WPRE), which enhances mRNA export and stability,^37^ was inserted downstream. The final construct (6.67 kb) was designated as the transgene.

#### Analysis of transgene insertion pattern

Transgene copy number was determined by Southern blotting, using *Dra*I-digested genomic DNA hybridized with a ^32^P-labeled probe corresponding to the EID1 region. Standards containing 1, 3, 10, and 30 copies of the transgene were included for comparison. DNA fragments were separated by agarose gel electrophoresis, transferred to Hybond-XL membranes, hybridized, and detected on X-ray film after one week of exposure at 4 °C. Insertion site analysis was performed using the RAISING method (Fasmac Co., Kanagawa, Japan).^38^ Briefly, single-stranded DNA was synthesized using transgene-specific primers, extended with adaptor sequences, and converted into double-stranded DNA for sequencing. The insertion site was mapped using the GRCm39 reference genome (GCF_000001635.27-RS_2024_02) and BLAST analysis.

#### ^18^F-FDG PET

PET imaging with ^18^F-fluorodeoxyglucose (^18^F-FDG) was performed as previously described to assess glucose uptake *in vivo*.^39^ Male mice (10 months of age) were fasted for >8 h and then exposed to either room temperature (25 °C) or cold (4 °C, 2 h) prior to imaging. After 1 h at the respective temperatures, mice were injected intravenously with ^18^F-FDG (5 MBq/mouse; *n* = 3 per group). PET scans were acquired 1 h later using an Inveon animal PET system (Siemens, Knoxville, TN) under isoflurane anesthesia, with a 10-min list-mode acquisition. Images were reconstructed with an OSEM3D/MAP algorithm (128 × 128 × 159 matrix) including attenuation correction. Tracer uptake in BAT was quantified as standardized uptake value (SUV).

#### Continuous core body temperature monitoring

Core body temperature was continuously measured using the DSI Anipill telemetry system (BodyCAP, Caen, France). Male mice (10 months of age; Tg, *n* = 5; Wt, *n* = 5) underwent intraperitoneal implantation of telemetry capsules under ketamine (80 mg/kg, Daiichi Sankyo Propharma Co., Ltd., Tokyo, Japan) and xylazine (10 mg/kg, Elanco, Greenfield, IN) anesthesia. Animals were allowed to recover for 6 days before experiments. Temperature was recorded every 15 min at room temperature (25 °C) for 3 days to verify circadian rhythmicity, and during 1 h of cold exposure (4 °C, 15:00–16:00), when diurnal variation is minimal. Capsule accuracy was validated against mercury thermometers before and after implantation.

#### Infrared thermography

Interscapular BAT surface temperature was measured noninvasively using an infrared camera (FLIR T530, Teledyne FLIR, Wilsonville, OR). Male mice (10 months of age; Tg, *n* = 4; Wt, *n* = 4) were acclimated at 25 °C for 1 h and then exposed to 4 °C for 1 h. Surface temperature between the scapulae was recorded before and after cold exposure and analyzed using FLIR Research Studio v2024.5.0 software.

#### Histological study

To observe the morphology of brown adipocytes, we performed HE staining on BAT. Interscapular BAT was harvested from Tg and Wt male mice (10 months of age; Tg, *n* = 4; Wt, *n* = 4) under deep anesthesia induced by intraperitoneal injection of ketamine and xylazine. After fixation in 4% paraformaldehyde, paraffin-embedded tissues were sectioned at a thickness of 5 μm and mounted on glass slides. The sections were then stained with hematoxylin followed by eosin according to standard protocols. Histological images were obtained using a light microscope (Eclipse E200, Nikon, Tokyo, Japan) at 400× magnification. For each section, five arbitrary areas of 1 mm^2^ each were selected, and the number of nuclei within each field was counted using ImageJ software (Version 1.54p, NIH, Bethesda, MD).^40^

#### RNA extraction

For DNA microarray and reverse transcription-PCR (RT-PCR) analyses, interscapular BAT was harvested from Tg and Wt male mice under deep anesthesia induced by intraperitoneal injection of ketamine and xylazine. Total RNA was extracted samples using the RNeasy kit (Qiagen, Hilden, Germany) and treated with DNase I (Qiagen) to remove contaminating genomic DNA.

#### DNA microarray

Mouse gene expression profiles (∼24,000 distinct genes) were analyzed using a 3D-Gene Mouse Oligo chip 24k (Toray Industries, Tokyo, Japan). Tg and Wt male mice were maintained for 1 h at either room temperature (25 °C) or cold exposure (4 °C). Total RNA (2.0 μg) extracted from BAT was labeled with cyanine 5 (Cy5), and hybridization was performed according to the manufacturer’s protocol. Hybridization signals were scanned using a 3D-Gene scanner (Toray Industries). Raw signal intensity for each spot was corrected by subtracting background values, and data were normalized using the global normalization method.^41^

#### Real-time PCR

Quantitative reverse transcription-PCR (qRT-PCR) was performed to validate the expression of genes identified by microarray analysis. Complementary DNA (cDNA) was synthesized from 1.0 μg of total RNA extracted from BAT using the High-Capacity RNA-to-cDNA kit (Applied Biosystems, Foster City, CA). qRT-PCR was carried out on an ABI StepOne Real-Time PCR system (Applied Biosystems) with TaqMan probes specific for mouse *Eid1* (Mn01337661_s1), *Ucp1* (Mm01244861_m1), *Adrb3* (Mm02601818_g1), peroxisome proliferator activated receptor γ (*Pparγ*, Mm00440940_m1), *Pgc-1α* (Mm01208835_m1), and human *EID1* (Hs00534885_s1). The mRNA expression was normalized to mouse glyceraldehyde 3-phosphate dehydrogenase (*Gapdh*) [Mm99999915_g1]. Relative gene expression was calculated using the 2^−ΔΔCT^ method.^42^ Expression levels in Tg mice were expressed relative to Wt controls at 25 °C (1 h), which were set to 1.0.

#### Western blotting

BAT tissue lysates were prepared in CelLytic MT buffer (Sigma-Aldrich, St. Louis, MO) supplemented with protease inhibitors. Proteins were separated by sodium dodecyl sulfate-polyacrylamide gel electrophoresis (SDS-PAGE), transferred to membranes, and probed with anti-UCP1 antibody (ab10983, lot no. 1050976-1, 1:5000; Abcam, Cambridge, UK). Horseradish peroxidase (HRP)-conjugated secondary antibody (Cytiva, Marlborough, MA) was used for detection. β-Actin (#4967, 1:1000; Cell Signaling Technology, Danvers, MA) served as a loading control. Protein bands were visualized using enhanced chemiluminescence (ECL Prime, Cytiva) and quantified with CS Analyzer 4 software (ATTO, Tokyo, Japan).

### Quantification and statistical analysis

#### Statistical analyses

Data are expressed as mean ± standard deviation (SD). One-way analysis of variance (ANOVA) followed by Dunn’s multiple-comparison test was used to analyze ^18^F-FDG uptake and expression of *Adrb3* and *Ucp1*. The Mann–Whitney *U* test was applied for *Eid1* expression, body weight, feed intake, core/BAT temperature and the number of nuclei in BAT. A *p* value <0.05 was considered statistically significant. Statistical analyses were performed using EZR software v1.60.^43^
